# A comparison of genotyping arrays

**DOI:** 10.1038/s41431-021-00917-7

**Published:** 2021-06-18

**Authors:** Joost A. M. Verlouw, Eva Clemens, Jard H. de Vries, Oliver Zolk, Annemieke J. M. H. Verkerk, Antoinette am Zehnhoff-Dinnesen, Carolina Medina-Gomez, Claudia Lanvers-Kaminsky, Fernando Rivadeneira, Thorsten Langer, Joyce B. J. van Meurs, Marry M. van den Heuvel-Eibrink, André G. Uitterlinden, Linda Broer

**Affiliations:** 1 Department of Internal Medicine, Erasmus MC, Rotterdam, The Netherlands; 2 Department of Pediatric Oncology, Erasmus MC, Rotterdam, The Netherlands; 3 Princess Máxima Center for Pediatric Oncology, Utrecht, The Netherlands; 4 Institute of Clinical Pharmacology, Brandenburg Medical School, Immanuel Klinik Rüdersdorf, Rüdersdorf, Germany; 5 Clinic of Phoniatrics and Pedaudiology, University Hospital Münster, Münster, Germany; 6 Department of Pediatric Hematology and Oncology, University Children’s Hospital, Muenster, Germany; 7 Department of Pediatric Oncology and Hematology, University Hospital for Children and Adolescents Lübeck, Lübeck, Germany; 8 Department of Epidemiology, Erasmus MC, Rotterdam, The Netherlands

**Keywords:** Genome-wide association studies, Genome-wide association studies, Clinical genetics, Genetics research, Genetic testing

## Abstract

Array technology to genotype single-nucleotide variants (SNVs) is widely used in genome-wide association studies (GWAS), clinical diagnostics, and linkage studies. Arrays have undergone a tremendous growth in both number and content over recent years making a comprehensive comparison all the more important. We have compared 28 genotyping arrays on their overall content, genome-wide coverage, imputation quality, presence of known GWAS loci, mtDNA variants and clinically relevant genes (i.e., American College of Medical Genetics (ACMG) actionable genes, pharmacogenetic genes, human leukocyte antigen (HLA) genes and SNV density). Our comparison shows that genome-wide coverage is highly correlated with the number of SNVs on the array but does not correlate with imputation quality, which is the main determinant of GWAS usability. Average imputation quality for all tested arrays was similar for European and African populations, indicating that this is not a good criterion for choosing a genotyping array. Rather, the additional content on the array, such as pharmacogenetics or HLA variants, should be the deciding factor. As the research question of a study will in large part determine which class of genes are of interest, there is not just one perfect array for all different research questions. This study can thus help as a guideline to determine which array best suits a study’s requirements.

## Introduction

Massive parallelization of solid support-based oligonucleotide hybridization approaches has led to the development of the most widely used platform for genetic analyses, i.e., genotyping arrays based on single-nucleotide variants (SNVs). Since 2005, genotyping arrays have been used for many applications, including clinical diagnostics of chromosomal abnormalities, genome-wide association studies (GWAS), fine mapping of known loci, and linkage studies. No matter the application, genotyping arrays measure from many hundreds of thousands to millions of SNVs across the genome, which are then assessed in relation to the research question or phenotype of interest. The number of available genotyping arrays is steadily increasing as more arrays are brought on the market, each with its own specific properties and content.

Of all the possible applications of genotyping arrays, traditionally its primary use has been GWAS, which has proven to be a successful research approach to discover genetic factors for complex diseases, toxicities, and traits [1–3]. An important factor in GWAS is “genome-wide coverage,” which is the extent to which SNVs on the array are evenly spread across the genome. Genome-wide coverage is a function of the number of genotyped SNVs and the capacity of these SNVs to tag adjacent (untyped) SNVs through patterns of linkage disequilibrium, which can be population-specific. For GWAS, one would ideally choose the genotyping array that provides the best genome-wide coverage for the studied population, however, these arrays tend to be more expensive. Of course, there are many other considerations for choosing a genotyping array such as the possibility to customize the content, the demands and wishes of a consortium or investigators (i.e., an interest in specific gene categories), and the imputation quality, all of which may influence the final decision.

As consumer genetics by companies like 23andMe has increased tremendously, the public need for including complex genetic information in the healthcare system is increasing [4]. In response, healthcare centers across the world have started programs to test the applicability of genotyping arrays in clinical practice for variant finding, pharmacogenetic testing, and polygenic risk scores (PRS) [5–7]. Due to this development, the presence of clinically applicable variants on arrays has become increasingly important.

In 2014, Ha et al. compared the performance of 12 arrays of different content released by Affymetrix/Thermofisher (hereafter called Affymetrix) and Illumina, for European, Asian, and African ancestries [8]. However, some of the investigated arrays are no longer available and new arrays have been launched since. In addition, only genome-wide coverage was considered by the authors, ignoring the coverage of specific frequently investigated genes and SNVs as well as the achieved quality when using the specific genotyped SNVs as template for imputation.

We, therefore, updated the previous study by Ha et al. and investigated all current genotyping arrays from Illumina and Affymetrix for their suitability for GWAS in the three major ethnicities (European, Asian, African). Also, we included in all comparisons two commonly used older and no longer available arrays (i.e., Human660Quad, Affy6.0) as reference. Furthermore, we included comparisons based on often studied gene categories with potential clinical applications (i.e., mitochondrial (mt)DNA, American College of Medical Genetics (ACMG) actionable genes [9], pharmacogenetic genes, and human leukocyte antigen (HLA) genes). In addition, we report the SNV density per gene and in 1-Kb/1-Mb windows, an influencing factor for specificity of copy-number variant (CNV) analyses, an important part of clinical screening. Our goal is to provide a comprehensive overview of the content of current genotyping arrays for both research and clinical applications. We will specifically not focus on how the design, costs, and automatization can influence the array choice from a laboratory standpoint. Nor will we focus on how collaborations (in a consortium) or previously available data can influence the choice of array, though an overview of array overlap is provided for those with existing genetic array data.

## Methods

### Array characteristics and genome-wide coverage

**Fig. 1:**
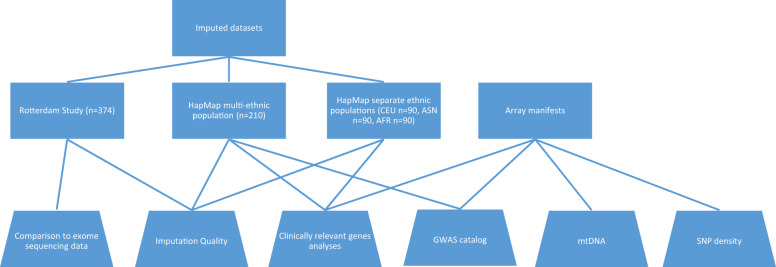
Flowchart of data used in this comparison. Rotterdam Study HRC1.1 imputed were used for comparison with sequencing data and imputation quality. HapMap and 1KGPp3v5-imputed genotypes were used for imputation quality, clinically relevant genes, and GWAS catalog analyses. Theoretical analyses such as mtDNA and SNV density used the array manifest files as basis.

We examined 28 arrays (10 from Affymetrix and 18 from Illumina), including the newest generation of genotyping arrays (Table 1). To examine the general characteristics of the included arrays, we downloaded the manifest files from the respective manufactures’ websites. These manifests were harmonized to the UCSC hg19 reference genome (and lifted over if required) and annotated using an Annovar-based pipeline to obtain detailed information on all loci [10]. The variants per array were grouped as autosomal, X-chromosomal and Y-chromosomal SNVs, exonic and splice-site variants, copy-number variations (CNVs), and mitochondrial DNA (mtDNA) variants. In addition, the overlap between arrays was calculated based on chromosome and position of each variant. The manifest files were further used for the majority of comparisons presented here. A short overview of the datasets and comparisons performed in each of the analyses described can be found in Fig. 1.

**Table 1:** General characteristics of investigated arrays.

			Content								
			Overall	SNPs/INDELs						CNVs	MT
Distributor	Array	ShortName^a^		Total	autosomal	X	Y	Exonic	Splice-site		
Illumina	Exome V1.1	Exome	242,901	242,682	237,436	5107	139	225,826	2082	0	219
Illumina	Immuno V2	Immuno	252,604	252,603	249,285	2115	1203	6840	280	0	1
Illumina	Cyto12	Cyto12	297,481	296,540	278,181	15,988	2371	5125	21	941	0
Illumina	Core	Core	298,930	298,725	288,675	8107	1943	33,327	5237	0	205
Illumina	DrugDev	DrugDev	475,233	475,035	459,659	13,405	1971	74,732	1025	0	198
Illumina	Onco	Onco	498,315	498,195	483,528	14,355	312	25,602	360	0	120
Illumina	PsychArray	Psych	570,100	569,789	554,577	13,890	1322	266,517	7451	0	311
Affymetrix	Axiom_GW_ASI	Axiom_ASI	630,191	629,957	610,467	17,268	2222	14,598	40	0	234
Illumina	660w-Quad	660w-Quad	652,132	587,527	573,493	14,015	19	11,189	37	64,484	121
Affymetrix	Axiom_GW_CHB	Axiom_CHB	657,615	657,520	632,267	24,273	980	10,283	282	0	95
Affymetrix	Axiom_NL	Axiom_NL	671,222	670,931	647,127	23,033	771	19,760	624	0	291
Affymetrix	Axiom_GW_EUR	Axiom_EUR	674,996	674,897	661,452	13,155	290	16,634	64	0	99
Illumina	OmniExpress	OmniExpress	715,322	715,322	695,789	18,166	1367	23,603	80	0	0
Illumina	GSAv1	GSAv1	618,540	618,406	601,120	16,100	1186	75,481	6231	0	134
Illumina	GSAv3	GSAv3	654,017	652,879	620,686	28,055	4138	75,481	2822	0	1138
Affymetrix	Axiom_LAT	Axiom_LAT	817,614	817,494	791,856	25,403	235	22,977	95	0	120
Affymetrix	Axiom_UKB	Axiom_UKB	845,485	845,131	823,234	21,084	813	137,657	4101	0	354
Illumina	CytoSNP850K	Cyto850	850,078	850,078	818,101	30,859	1118	37,793	1025	0	0
Illumina	OmniZhongHua	OmniZH	899,502	899,381	873,429	23,935	2017	23,167	59	0	121
Affymetrix	PMRA	PMRA	920,744	920,520	883,265	37,246	9	77,781	6536	0	224
Affymetrix	PMDA	PMDA	921,664	920,950	857,925	62,577	448	51,811	3337	0	714
Affymetrix	Affy6.0	Affy6.0	933,122	932,711	893,949	37,902	860	8966	33	0	411
Illumina	MultiEthnic-AMR/AFR	Multi_AFR	1,426,177	1,425,533	1,380,255	42,918	2360	357,838	4922	0	644
Illumina	MultiEthnic-EUR/ASN	Multi_EUR	1,474,463	1,473,819	1,432,449	39,772	1598	358,382	5062	0	644
Illumina	MultiEthnic-Global	Global	1,768,335	1,767,356	1,707,340	56,079	3937	399,721	10,325	0	979
Affymetrix	Axiom_GW_PanAFR	Axiom_AFR	2,268,172	2,267,890	2,199,268	65,975	2647	21,954	100	0	282
Illumina	Omni2.5	Omni2.5	2,435,293	2,435,200	2,376,441	57,178	1581	70,425	201	0	93
Illumina	Omni5	Omni5	4,082,393	4,082,109	3,965,887	113,724	2498	128,327	5298	17	267

^a^Manufacturers’ array names were shortened to improve readability, mostly by compressing population names, or removing version names if irrelevant or superfluous. Four arrays have an acronym as name: the Global Screening Array (GSA, both version), the Precision Medicine Research Array (PMRA), and the Precision Medicine Diversity Array (PMDA).

Genome-wide coverage was defined as the fraction of all SNVs in the 1000 Genomes Project phase 3 version 5 (1KGPp3v5) reference that can be captured by the array [11, 12]. The reference panel was divided in three separate groups based on genetic background, namely European (*n* = 379), Asian (*n* = 286), and African ancestry (*n* = 246). Genome-wide coverage was calculated using the equation defined by Li et al. [12]

$${{\mathbf{CR}}} = \frac{{\frac{{\boldsymbol{L}}}{{{\boldsymbol{R}} - {\boldsymbol{T}}}} \ast \left( {{\boldsymbol{G}} - {\boldsymbol{T}}} \right) + {\boldsymbol{T}}}}{{\boldsymbol{G}}},$$

in which *L* denotes the number of SNVs tagged by SNVs on the array (*r*^2^ > 0.8), *R* denotes the total number of SNVs in the reference panel, and *T* denotes the total number of variants on the array. Whereas, *G* represents an estimate of the total number of SNVs in the human genome. Currently the number of validated SNVs with a minor allele frequency (MAF) > 1% in the NCBI database is ~19 million. For each previously mentioned genetic background, the reference panel was filtered to keep variants with a MAF of 1% or higher. The majority of investigated arrays were designed to capture variants with a MAF > 1% of their specific design populations only and comparing the arrays on their genome-wide coverage of lower MAF would therefore not be appropriate. After filtering, the reference set contained 7,295,404 SNVs for European ancestry (EUR), 6,654,452 SNVs for Asian ancestry (ASN), and 10,924,094 SNVs for African ancestry (AFR). Of these, 85% in EUR, 88% in ASN, and 66% in AFR could be tagged by another variant in the 1KGPp3v5 set with a LD of *r*^2^ > 0.8 within each dataset.

### Imputations

Using the manifest files to compare arrays can introduce biases as not all variants included in these files will actually be included in the genotyped output. This is caused by variants being excluded during the generation of genotypes due to low cluster quality (Supplementary Fig. 1).

To check for potential manifest bias, we additionally investigated the genotyping rate and imputation quality across genetic backgrounds, for which Illumina and Affymetrix kindly provided raw genotype data of HapMap samples (*N* = 210) for a subset of the investigated arrays (Core, OmniExpress, GSAv1, PMRA, Global and Omni2.5). As the HapMap samples are also included in the 1KGPp3v5 reference panel, an imputation bias may be introduced due to overfitting of imputed genotypes to the haplotypes of the same samples. To exclude such an imputation bias, we additionally genotyped 374 European-ancestry women from the Rotterdam Study (RS) [13] on four different genotyping arrays (550K, GSAv1, PMRA and Omni5). Before imputations, standard quality control (i.e., SNV and sample call rate <97.5%, Hardy–Weinberg Equilibrium <1 × 10^−7^, or excess heterozygosity) was performed on each dataset [14]. Imputations were performed using the Michigan Imputation Server [15]. For the imputations of HapMap samples, the 1KGPp3v5 reference panel [11] was chosen, rather than the larger and newer HRC r1.1 reference panel [16] as the latter contains an overrepresentation of European haplotypes, which might influence results for other genetic backgrounds. The three genetic backgrounds of the HapMap samples were imputed separately (EUR (*N* = 90), AFR (*N* = 90), ASN (*N* = 90)) as well as in a combined dataset (*N* = 210). For the imputations of the set of the RS, HRC r1.1 was chosen instead, as all genotyped samples are women from North-European ancestry. All chromosomes (except Y) were imputed, and the imputation quality (*R*^2^) was extracted. Any variant with a *R*^2^ lower than 0.3 was considered to be of low quality, while any variant with a *R*^2^ higher than 0.8 was considered to be of high quality. Variants with a *R*^2^ between 0.3 and 0.8 were designated as medium quality. Furthermore, four MAF bins were created (<0.5%, 0.5–1%; 1–5%; >5%) to differentiate between ultra-rare, rare, low frequency and common variants, respectively. To evaluate the accuracy of these bins, the number of variants for each bin and average concordance with WES data of the same samples was calculated per chromosome using vcftools diff-indv-discordance [17]. Due to the overabundance of reference alleles skewing the concordance metric, only genotypes with variant alleles were considered for the ultra-rare and rare categories, by setting homozygous reference genotypes (0/0) to missing (./.). After this validation for variants mostly in coding sequences, the percentage of concordant low-, medium-, and high-quality variants within each MAF bin was compared across arrays.

For all further analyses evaluating the imputation quality of arrays, the combined HapMap (EUR+ASN+AFR, *N* = 210) set was used. For the different gene categories investigated further, only variants with high imputation quality (*R*^2^ > 0.8) were considered to be callable by imputation rather than the standard cutoff of *R*^2^ higher than 0.3 used in GWAS (Fig. 1). The reason for this more stringent imputation quality cutoff is the potential usability of these arrays in clinical practice where high accuracy of genotype calls is required and will be used throughout this study.

### GWAS and PRS

To investigate the usability of arrays for GWAS as well as the inclusion of PRS in clinical practice, we assessed the presence of known GWAS markers available on the array or after imputations with a good imputation quality. Freeze 2020-10-07 of the GWAS catalog was downloaded (212,730 records) and filtered based on the following criteria: effect size (OR) and risk allele frequency reported in the database, *p* value ≤ 5 × 10^−8^, duplicate records (i.e., same phenotype-loci association in more than one study), case–control studies, disease phenotypes (e.g., hair color, eye color loci were removed), and impact factor (2019) of the journal >5 [2]. This filtering resulted in 6054 markers to be investigated. In addition, the known variants for APOE*4 associated with Alzheimer’s disease (rs7412; NC_000019.9:g.45412079C>T and rs429358; NC_000019.9:g.45411941T>C) were added. APOE*4 in GWAS is tagged by a *TOMM40* SNV [18]; however, as the actual causal APOE*4 variants are of such importance to the field, we decided to add them to this investigation. In total, we therefore considered 6056 GWAS markers.

### Mitochondrial DNA (mtDNA)

The online MITOMAP resource was used to divide the mtDNA variants into 75 functional loci (i.e., genes as well as other features such as important binding sites) [19]. For each locus, the number of genotyped variants on the array was extracted from the manifest files. As the mtDNA loci overlap, also the total number of mtDNA variants was investigated.

### Current clinical applications

#### ACMG actionable genes

The total number of genotyped variants in the 59 so-called “actionable genes,” as proposed by the ACMG [9], were extracted from the array manifest files. The ACMG recommends the use of ClinVar variant status for the purpose of checking pathogenicity; however, this status is not always correct nor is it set in stone [20]. In our study, we have thus translated this definition to any variant in the ACMG genes with a Combined Annotation-Dependent Depletion (CADD) score higher than 20 (i.e., top 1% of most damaging variants), which were considered to be potentially damaging [21].

#### Pharmacogenetic genes

A list of 388 genes involved in pharmacokinetics and pharmacodynamics was acquired by combining the list of pharmacogenomics biomarkers in drug labeling published by the U.S. Food and Drug Administration (http://www.fda.gov/Drugs/ScienceResearch/ResearchAreas/Pharmacogenetics/ucm083378.htm) and the list of pharmacogenetics markers published by the Pharmacogenomics Knowledge Base (PharmGKB) [22]. The presence of variants in these genes on the arrays was determined using the manifest files.

Several genes from the cytochrome P450 (CYP) complex were further investigated for the presence of the so-called star(*)-alleles [23] on the arrays and/or imputed data, based on their documented tag SNVs. The entire haplotype, the *-allele was based on, should be present. As the number of *-alleles per CYP genes differ, the percentage of called *-alleles per gene was assessed. Additional *-alleles for other pharmacogenetic genes are known from databases such as the PharmGKB [22]; however, these are outside the scope of this study.

#### HLA genes

Tag SNVs in six HLA genes encoding HLA class I and HLA class II serotypes were used to determine to what extent HLA types for EUR, ASN, and AFR ancestries could be identified by genotyping arrays [24]. HLA serotypes are often identified by *-allele codes. We considered an HLA *-allele to be identifiable by an array if all tag SNVs for that allele were present on the array. As the number of *-alleles per HLA gene differs, the percentage of called *-alleles per gene was assessed. In addition, the tag SNVs for HLA *-alleles differ between genetic ancestries. Therefore, percentages of *-alleles per gene were assessed separately for each genetic ancestry.

#### SNV density

To determine the specificity of the arrays for calling structural variant (SV) or CNV analysis, the density of SNVs on the array, both in exonic regions and overall, was used as a proxy. A higher density of variants would result in a better resolution to define where an SV or CNV starts and ends. To calculate the overall density of SNVs within coding regions of genes, a custom perl script was used. The total length of coding sequence per gene was divided by the number of SNVs + 1 resulting in a mean distance between variants within a gene (*n* = 20,445 genes, GENCODE (v35), APPRIS principal transcripts). In addition, average SNV density and standard deviation (SD) per 1-Kb and 1-Mb windows were calculated over the entire genome.

## Results

### Array characteristics and genome-wide coverage

In total, we examined 28 arrays (10 from Affymetrix and 18 from Illumina), including the latest generation of genotyping arrays, the GSA (v1 and v3), the PMRA, and the PMDA (Table 1). The overall number of markers on the arrays ranges from ~240K (Exome) to ~4M variants (Omni5). Most of the currently available arrays have none or only a few dedicated CNV markers (i.e., intensity only probes) on the array. In comparison, the 660w-Quad array had ~65K CNV markers included in its design. The number of variants mapping to the Y-chromosome and mtDNA, unlike the X-chromosome, does not necessarily increase with the overall number of variants on the array (Table 1). Newer arrays (e.g., GSA, PMRA, PMDA, Global) have more exonic and splice-site variants included in the design compared to older ones. The two exceptions to this are the older Exome (exonic variants = 225,826, 93% of total content) and the disease-specific Psych (variants = 266,517, 47% of total content) arrays.

Though not discussed in detail in this article, the backwards compatibility of arrays can be an important deciding factor for studies who have already genotyped a large percentage of their study population in the past. We have therefore included an array overlap table in Supplementary Table 1.

The genome-wide coverage of the investigated arrays ranges from 2 to 84% in EUR and increases with the number of variants on the array (Supplementary Fig. 2A). The genome-wide coverage is higher in ASN (3–100%; Supplementary Fig. 2B) and lower in AFR (2–40%; Supplementary Fig. 2C) ancestries. The Axiom_AFR achieves a higher genome-wide coverage in EUR (47%) and ASN (53%) ancestries compared to either the Axiom_EUR (19%) or Axiom_ASI (23%) arrays for their respective ancestries. This can be explained by the expected improvement of genome-wide coverage with a larger number of variants on the array, independent of the targeted study population. Noticeably, the trend for higher coverage with more variants on the array is disrupted for the latest arrays (i.e., GSAv1, GSAv3, PMRA, and PMDA) as these seem to have been designed with imputation quality in mind rather than genome-wide coverage.

### Imputations

**Fig. 2:**
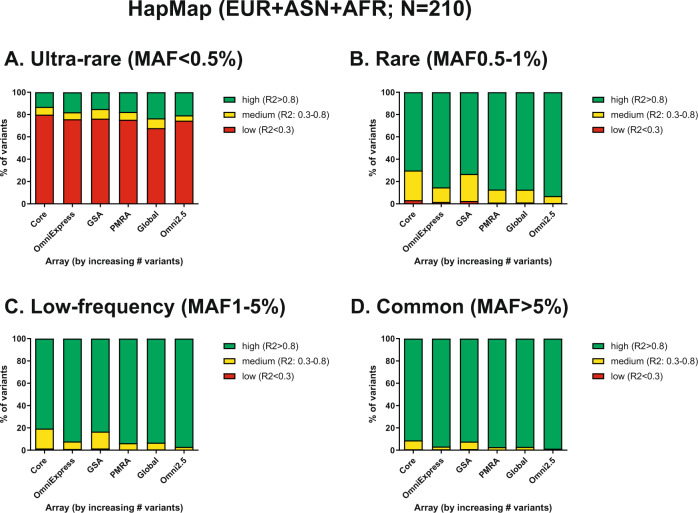
Imputation quality (1KGPp3v5) of HapMap samples for six different arrays, stratified by minor allele frequency. **A** describes the imputation quality for the ultra-rare variants (MAF<0.5%), **B** describes the imputation quality for the rare variant bin (MAF 0.5–1%. **C** describes the imputation quality for the low-frequency variants (MAF 1–5%) and **D** describes the imputation quality for common variants (MAF >5%). All bins are coloured by high (green), medium (yellow) or low (red) imputation quality *R*^2^, >0.8, 0.3–0.8 and <0.3 respectively.

For the HapMap samples, six arrays were evaluated. A general trend of little difference in imputation quality for the various arrays was observed for all genetic backgrounds (Supplementary Figs. 3–5). However, in EUR ancestry samples, the Global array was better at calling ultra-rare variants (MAF < 0.5%) than any other array (4.3% compared to <0.1%). Interestingly, imputation of ASN samples resulted in lower quality than imputation of AFR samples, especially for rare and low-frequency variants. When imputing the AFR, ASN, and EUR samples in one set, the imputation quality markedly increased (Fig. 2), especially for ultra-rare (MAF < 0.5%) and rare (MAF 0.5–1%) variants, probably due to the increased number of input haplotypes. When comparing the GSAv1 and PMRA, the PMRA had slightly higher imputation quality for common and low-frequency variants. This difference was more pronounced for rare (MAF 0.5–1%) variants (87.2% of high quality compared to 73.3% for GSAv1).

Based on a starting set of 511,992 (550K), 3066,764 (Omni5), 516,598 (GSAv1), and 472,151 (PMRA) SNVs before HRC r1.1 imputation, all datasets from RS imputation contained roughly 40 million variants after imputation. Of these, around 31 million (79%) were in the ultra-rare category (MAF < 0.5%), 1.2 million (3%) rare (MAF 0.5–1%), 2.4 million (6%) low frequency (MAF 1–5%), and around 5.5 million (14%) were common variants (MAF > 5%). Comparing these imputation sets to the WES dataset of RS samples resulted in varying amounts of concordance, but a similar number of overlapping variants (around 225k per dataset), with the exception of the Omni5 at 232k overlapping variants (Supplementary Fig. 6). More common SNVs are generally imputed better across all arrays than rare SNVs as can be seen by the number of SNVs in each of the bins. Interestingly, concordance is high for all sets in the high imputation quality (*R*^2^ > 0.8) bins, while concordance drops sharply in lower (*R*^2^ < 0.8) bins. When evaluating the percentage of high-, medium-, and low-quality imputed variants of the above-mentioned datasets, all four tested arrays performed very similarly (Supplementary Fig. 7). Only for rare and low-frequency variants the Omni5 array had better imputation quality compared to arrays with a lower number of variants.

### GWAS and PRS

The percentage of variants from the GWAS catalog (6,056 variants; see “Methods”) called by the arrays either by direct genotyping or after imputations with good imputation quality (*R*^2^ > 0.8), ranges from 10.6 (Cyto12) to 52.0% (Omni5); Supplementary Fig. 8). After imputations, all tested arrays had a high coverage of known GWAS markers with high imputation quality (>90% covered) with minimal differences per array.

### Mitochondrial DNA (mtDNA)

The number of variants for the mtDNA does not increase with the overall number of variants on the array (Table 1 and Supplementary Fig. 9A). In fact, several arrays (i.e., Cyto12, OmniExpress, and Cyto850) do not contain mtDNA variants at all, while the Immuno array only has one variant for the NADH-ubiquinone oxidoreductase chain 1 (mt-ND1). In general, Illumina arrays appear to have more mtDNA variants per mitochondrial feature than Affymetrix arrays (Supplementary Fig. 9B).

### Current clinical applications

#### ACMG actionable genes

An overall trend was observed with an increasing number of variants on the array resulting in more variants located inside the ACMG actionable genes (Supplementary Fig. 9C). When comparing the older arrays (Affy6.0 and 660w-Quad) to more recent and currently available arrays, the new arrays of similar size generally have more variants dedicated to the ACMG actionable genes.

**Fig. 3:**
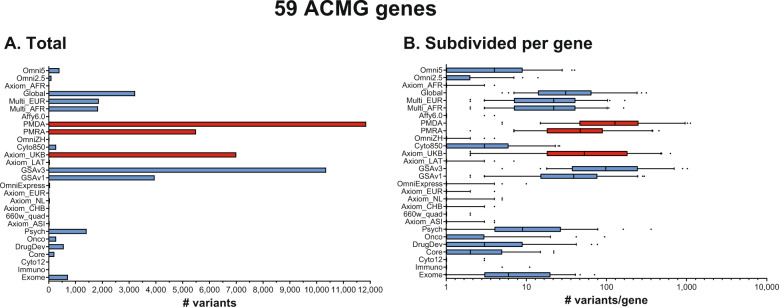
Number of variants located in the ACMG actionable genes with a CADD > 20, indicating possibly damaging variants. Arrays are ordered by size with the smallest array at the bottom. Arrays in blue are from Illumina, while arrays in red are from Affymetrix. Bar chart (**A**) represents the total number of ACMG-overlapping variants present on the array. The box plots (**B**) represent the median and interquartile range.

When taking the CADD score into account, the trend with the size of the array is no longer observed (Fig. 3A and Supplementary Table 2). Instead, newer arrays have more “proxy-deleterious” variants (variants with a high CADD score) in the ACMG actionable genes in their design. The highest number of these variants is found in the PMDA (11,851), PMRA (5,500), and Axiom_UKB (7,001) for Affymetrix, and GSAv3 (10,353) and GSAv1 (3,954) for Illumina. Observing the distribution of proxy-deleterious variants for these arrays over all ACMG genes provides a similar picture: PMDA (median 128), PMRA (median 47), Axiom_UKB (median 53), and GSAv3 (median 98), GSAv1 (median 39) (Fig. 3B and Supplementary Table 2).

#### Pharmacogenetic genes

**Fig. 4:**
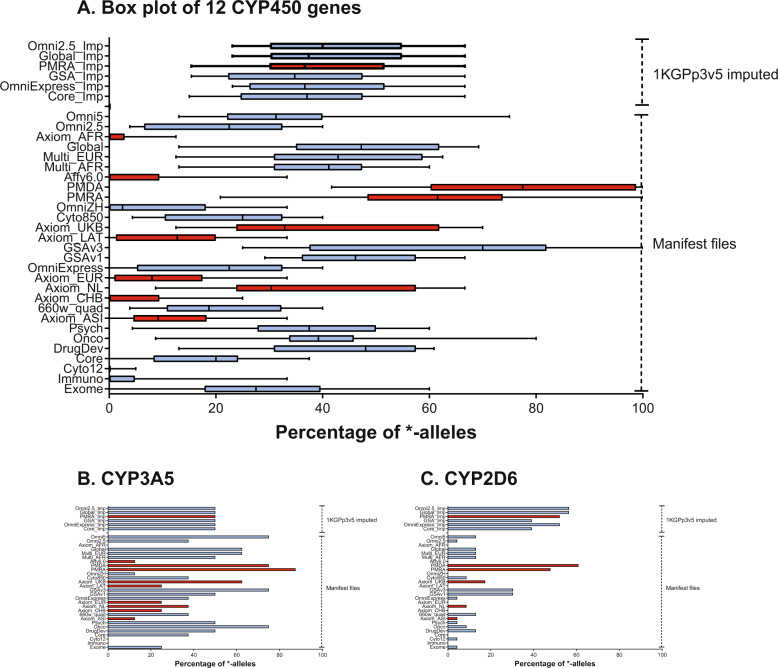
Percentage of CYP450 *-alleles called. Arrays are ordered on size with the smallest array on the bottom. At the top are imputed datasets. Arrays in blue are from Illumina, while arrays in red are from Affymetrix. The box plots (**A**) represent the median and interquartile range. Bar charts (**B**) and (**C**) represent two examples of *-allele coverage of CYP450 genes.

A trend can be observed where newer arrays have more variants dedicated to pharmacogenetics (Supplementary Fig. 9D). However, known pharmacogenetics marker *-alleles (star alleles) are very specific and need a certain SNV or set of SNVs to be identified. As such, most of the variants in the pharmacogenetic genes can be discarded during pharmacogenetic analysis. In total 186 *-alleles were evaluated for 12 CYP450 genes. Especially newer arrays such as GSAv1, GSAv3, PMRA, or PMDA scored well in covering *-alleles of CYP450 genes (Fig. 4A). After imputations, the coverage of the *-alleles generally decreases. This is caused by the low allele frequency (MAF < 0.5%) of many of these variants, which get excluded during imputation. In addition, there is a broad variation in the ability to call the *-alleles of different CYP450 genes (Supplementary Fig. 10 and Supplementary Table 3). For example, Axiom_UKB calls ~60% of known *CYP3A5* *-alleles (Fig. 4B), while only calling ~20% of known *CYP2D6* *-alleles (Fig. 4C). The same trend holds for these two genes on most other arrays.

#### HLA genes

**Fig. 5:**
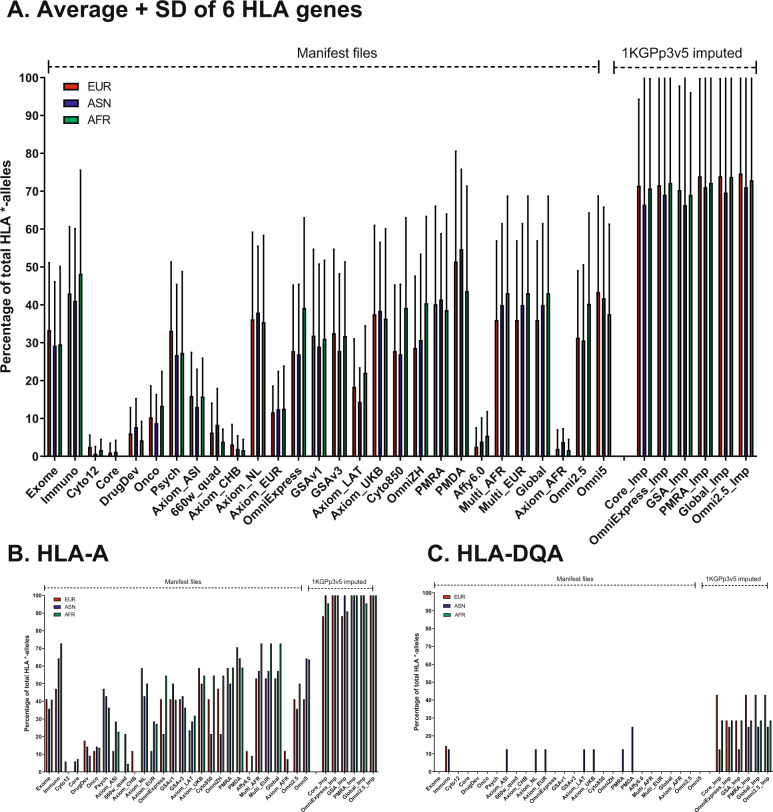
Percentage of HLA *-alleles called. Percentages shown separately for the three ethnicities as different tag SNPs are correlated with HLA classes per ethnicity. Arrays are ordered on size with the smallest array shown on the left. Imputed datasets are placed on the right. Bar chart (**A**) describes the average and SD coverage of *-alleles over the 6 HLA genes, while charts (**B**) and (**C**) show the difference in coverage for a class I and class II HLA gene.

Despite the large number of variants both Illumina and Affymetrix assign to the HLA region in general, on average only up to ~45% of the known HLA *-alleles can be genotyped even with the newest arrays (Fig. 5A). Also, the Immuno and Psych arrays had similarly high coverage of HLA types. This can be explained by the targeted design of these arrays for specific diseases and known associations for these diseases. There is a strikingly high coverage of HLA after imputations, with up to 100% of the population being covered, probably due to the very-high LD in this region. There is a notable difference between Class I and Class II HLA types, with Class I types being covered much better compared to Class II types (Supplementary Fig. 11 and Supplementary Table 4). For example, *HLA-A* (Fig. 5B) has ~50% of its known *-alleles called on most arrays, while *HLA-DQA* (Fig. 5C) is not called at all. After imputations, the percentage of called *-alleles in Class I HLA types is almost 100% while it is only ~50% of Class II HLA types.

#### SNV density

The average distance between SNVs per coding regions of gene per array was plotted to compare the various arrays on SNV density. A lower distance between SNVs meant a higher density for that specific region (Supplementary Fig. 12). There seemed to be no direct trend for array size and SNV distance, since even larger arrays such as the Axiom_AFR had similar average distances as smaller arrays such as the OmniExpress. The largest array, the Omni5, had a lower density in coding regions than a specialized array like the Psych array, though the Psych array covers fewer genes in total. However, the newest arrays (GSA and PMRA) did show higher coding SNV density when compared to older arrays. Focus on exonic variation was also clearly visible in the number of genes containing at least one SNV, where the specialized Psych array, as well as the Global, Multi_EUR, and Multi_AFR had a high number of SNVs located within coding regions relative to their size. Interestingly, Affymetrix arrays design seemed to have less resolution to detect exonic variants, with only the two most recent arrays (Axiom_UKB and PMRA) having a high number of exonic SNVs in addition to a low mean distance. Studying the overall genomic density through 1-Kb and 1-Mb windows shows a slightly different picture with larger arrays having a higher overall density regardless of design type as can be seen in (Supplementary Table 5).

## Discussions

### Array characteristics and coverage

The currently available arrays differ quite a lot in the number of markers on the arrays and subsequently genome-wide coverage. Interestingly, the Axiom array created for ASN ancestry achieved a similar genome-wide coverage in EUR ancestry as the array specifically created for EUR ancestry. As discussed below, this increase in genome-wide coverage with a larger number of variants does not necessarily translate into a higher imputation quality for most recent arrays. Therefore, we propose to abandon genome-wide coverage as a selection criterion for array choice. In addition, there is a trend toward excluding CNV markers, with most current arrays containing no CNV markers. Instead, the intensities of SNV markers are now used to determine insertions and deletions in the samples (discussed in more detail in Coding SNV density). Also, it should be noted that there can be large differences in SNV content even between consecutive arrays of the same manufacturer with little backward compatibility. As such combining cohorts for case–control studies, or follow-up data generated on a different platform can be a challenge in practice.

### Imputations

While the amount of input variants for imputations for each of the arrays differs after imputation, the actual imputation quality of all MAF categories is roughly the same regardless of the input source. This suggests that the number of variants on the array, and thus the genome-wide coverage, do not influence imputation quality. However, this will not always be the case, as an uneven spread of variants on an array over the genome would cause some linkage blocks to not be covered properly. As a result, linkage for these chunks would not be calculated and variants would not be determined on these chunks as implemented in the Michigan Imputation Server [15]. As such, an even spread of variants over the genome is very important for the imputation results. However, as most current arrays are made with GWAS in mind, we do not expect this to be a problem for current and future arrays. The only exceptions to this rule, currently, would be the Exome, Immuno, Cyto12, DrugDev, Psych, Onco, and Cyto850 arrays, which were made focusing on exons (Exome), specific diseases (Immuno, Psych, and Onco), pharmacogenetics (DrugDev), or CNV calling (Cyto12 and Cyto850) instead. We do see that the imputation quality of the Omni5 array (~4M variants) is slightly better compared to the other tested arrays. However, due to the high price of this array, resulting in fewer samples to be genotyped for the same budget, we feel that this slight gain in accuracy is not worth the cost. We conclude that, as long as an array was made with GWAS in mind, the choice of array should not depend on the number of variants on the array.

When comparing the results of the imputation of several arrays against a different technique such as WES, we noticed that only a small percentage of the imputed variants is located within the human exome. However, common (MAF > 0.05) and low-frequency (MAF 0.05–0.01) SNVs are imputed very well, as can be deferred from the large number of SNVs in high imputation quality (*R*^2^ > 0.8) bins, when compared to the lower quality bins (*R*^2^ 0.3–0.8 and *R*^2^ < 0.3). Based on the concordance with WES genotypes, we can conclude that using a strict filter of *R*^2^ > 0.8 yields genotypes, which are true in most cases (average concordance: 98.9% for common, 99.6% for low frequency, 97.9% for rare, and 96.6% for ultra-rare variants). This is important if imputed data are to be used in a predictive and/or clinical setting.

### GWAS and PRS

As expected, known GWAS loci as summarized in the GWAS catalog are better covered by newer arrays. This catalog is often used in the design of these arrays, which completely explains this phenomenon. After imputations, however, all arrays have high coverage of known GWAS loci. Therefore, whether interested in GWAS or in designing PRS for research purposes, there is no best array to choose. Going for a cheap, up-to-date GWAS oriented array will provide you with the most coverage available. However, if one is interested in implementing PRS into a clinical setting imputations may not be accepted. In this setting, a recent GWAS array with added customized variants is most advantageous. Both the PMDA and GSA arrays are customizable and would be suited for this purpose.

### Mitochondrial DNA (mtDNA)

The number of mtDNA variants included in arrays is not related to the overall number of variants on the arrays. mtDNA genetic variants were originally included for QC purposes as they can be used to determine genetic ancestry [25]. However, these variants have since been used for genotype–phenotype associations as well. Both the haplogroups themselves [26–28] and individual variants [27, 29, 30] have been found associated with several phenotypes. However, unlike nuclear DNA, mtDNA is primarily maternally inherited and present in multiple copies per cell [31]. Due to this, variants in mtDNA within an individual follow the laws of population genetics, rather than Mendelian genetics [32]. In addition, old beliefs that humans are homoplasmic (i.e., all mtDNA molecules are identical) at birth have changed and a more heteroplasmic model (i.e., multiple different mtDNA molecules) is now accepted in the scientific community, with ~25% of individuals being heteroplasmic at birth [32, 33]. Nevertheless, it is currently unknown if genotyping arrays are sensitive enough to pick up low-grade heteroplasmy as present in the general population. On top of these complexities, mtDNA has a very-high mutation rate resulting in higher levels of mtDNA diversity as individuals age [34]. Therefore, the question may not be how many mtDNA variants are present on the arrays, but how to analyze them properly. As a result, we believe that, currently, the number of mtDNA variants on the array should not be used as a criterion for array choice. This may change in the future as new methods are developed to analyze the mtDNA variants.

### Current clinical applications

#### ACMG actionable genes

The ACMG published a list of 59 genes in which variants could lead to severe outcomes, but which are medically actionable [9]. Though this list was originally meant for reporting possible additional variants in these genes in patients undergoing clinical sequencing for other purposes, the list has also had an influence on array design, with new arrays including more variants in these genes in their designs. It should be noted that for many genes in the recommendations of the ACMG, it is stated that even expected pathogenic variants (i.e., even variants where the clinical status has not been proven yet) should be reported to the patient [9]. The main database that is to be used for this purpose according to the ACMG is the ClinVar variant status. However, in a previous study, we have determined that this variant status is not a stable source, with variants changing pathogenicity status often [20]. To prevent the observer bias in the ClinVar database, we have thus translated this definition to any variant in the ACMG genes with a CADD > 20, indicating that the variants are in the top 1% of most damaging variants overall [21]. A surprisingly large number of variants with such high CADD scores is included in the array designs of the Axiom_UKB, PMRA, and GSAv1 arrays. This is even more prominently the case in the PMDA and GSAv3 arrays, which can be considered to be updated versions of the PMRA and GSAv1 arrays, respectively. This is good news for the possibility of using arrays for variant screening purposes. For research purposes, however, this may not be desirable as reporting back such variants to healthy participants can be problematic, especially as 1 in 38 healthy individuals carries at least one likely pathogenic variant [35]. Some older arrays, like the OmniExpress, have little to no variants with a high CADD score in the ACMG genes included in their design, which can make these arrays decent substitutes.

#### Pharmacogenetic genes

It is difficult to create a complete pharmacogenetic profile of a sample regardless of which of the evaluated arrays is used. There is, however, a clear trend in newer arrays of including more pharmacogenetic content. A trend has only increased in intensity as observed when comparing the GSAv1 and PMRA with their respective successor arrays, the GSAv3 and the PMDA. Still, even the newest arrays do not cover all *-alleles and show a wide variability in their ability to call a specific pharmacogenetic gene. This can mostly be explained by the complex nature of certain pharmacogenetic genes (e.g., CYP2D6) for which designing probes is hampered by the complexity of the region as well as pseudogene interference [36]. In addition, some CYP genes are known to have large CNVs (i.e., gene duplications and gene deletions). Calling these requires CNV analysis, which is outside of the scope of this study. Furthermore, the presence of the CNVs makes calling other variants more difficult as these may also be present in one, two, three, or more copies. Affymetrix has therefore implemented a copy number aware calling algorithm for their newest (i.e., PMRA and PMDA) arrays, as well as an automated SNV to *-allele translation algorithm. It should be noted that currently the calling of *-alleles using arrays is purely on a theoretical basis and that the correctness of made pharmacogenetic calls has not been investigated here. However, if one is interested in pharmacogenetic calling for research and/or clinical testing, currently the best array on the market is the PMDA array from Affymetrix, with the GSAv3 array from Illumina being a close second.

#### HLA genes

On average, only about 45% of the HLA *-alleles can be genotyped with arrays. There is a striking difference between HLA class I and HLA class II types, with HLA Class I being covered better than Class II. This can be explained by large structural variations in the HLA region, particularly in the HLA Class II region [37]. Even so, the HLA region is associated with many different traits in GWAS studies as reviewed in ref. [38] and is relevant also in organ transplantation [39]. Currently, the best HLA typing method is a sequence-based technique [40]. As Class I HLA types are most important for transplantations [39] and these HLA types are covered well, up to 100% after imputations, recent arrays, in particular the PMDA, might be an interesting and cheap alternative. It should be noted that multiple specialized HLA imputation tools exist and one of these is implemented in an Affymetrix HLA calling tool, which improves the calling of HLA types even more than using the tagging SNVs shown here [41]. Whether this is a clinically viable option or not needs to be validated.

#### SNV density

Overall, most arrays have similar average distances between SNVs within the coding regions of genes. However, newer arrays such as the GSA or the PMRA show a higher focus on exonic variants, possibly due to the clinical relevance of these variants in fields such as pharmacogenetics. The lack of a high exonic content in older arrays can be at least partially explained by their focus on GWAS, which requires an even spread of variants across the genome instead of focus on the coding regions. This effect is also visible in the density results for 1-Kb and 1-Mb windows, where a higher number of genotyped variants directly correlates to the overall density of variants on the genome.

### Overall conclusion

While the comparison of arrays we performed here does not cover the complete scope of all applications of genomic microarray technology, this study considers the most commonly used applications in-depth. Overall, we conclude that differences between arrays are mostly small between arrays of a similar age and that the choice of array is very dependent on the research question. As discussed in the individual sections above, the PMDA array from Affymetrix is particularly rich in ACMG actionable gene variants, pharmacogenetic content, and the ability to call HLA types. The Illumina counterpart that the GSAv3 also scores high in all three categories.

We have also seen that currently the new updated arrays being developed by Illumina and Affymetrix have an even stronger focus on these potentially clinical applications of arrays, which bodes well for future use of array technology in a clinical setting as first screening algorithm. It also means that we can safely assume that new arrays arriving after printing of this study will have further developed in these fields compared to their predecessors.

For a practical example on array choice in a new study, we would like to focus on the PanCareLife study [42, 43]. In the PanCareLife study, we were interested in both pharmacogenetics as well as GWAS, in order to study the late-life effects of treatment in childhood cancer survivors. As the PMRA array was not available at the time, we chose the GSAv1 array for this study because of its good coverage of pharmacogenetic variation and good GWAS backbone. If this same study were designed now, the GSAv3 or PMDA array would have been chosen instead. Similar decisions can be made for other studies using the comparison as provided in this study.

## Disclaimer

Even though genotyping data for the HapMap samples were provided by Affymetrix and Illumina, neither company had any say in the design or execution of the study. The results of our study were shared with Affymetrix and Illumina only after completion of the study and manuscript.

## Supplementary information

Supplementary figure legends

Supplementary Figure 1

Supplementary Figure 2

Supplementary Figure 3

Supplementary Figure 4

Supplementary Figure 5

Supplementary Figure 6

Supplementary Figure 7

Supplementary Figure 8

Supplementary Figure 9

Supplementary Figure 10a

Supplementary Figure 10b

Supplementary Figure 11

Supplementary Figure 12

Supplementary Table 1

Supplementary Table 2

Supplementary Table 3

Supplementary Table 4

Supplementary Table 5

## Data Availability

Original array manifest files used for the theoretical comparisons are available on the manufacturers’ websites. Annotated (and lifted-over) variant files used for the theoretical comparisons for each array are available in.bed format and can be downloaded from: https://github.com/jverlouw/ArrayComparisonData. Raw genotype data of the HapMap samples for the Core, OmniExpress, GSAv1, Global, Omni2.5, and PMRA arrays should be requested through Illumina and Thermofisher for their respective arrays. Due to legal and ethical restrictions, individual-level genotype data of Rotterdam Study (RS) participants cannot be made publically available in a managed-access database. Data are available on reasonable request to the Rotterdam Study management team (chair M. Arfan Ikram, m.a.ikram@erasmusmc.nl) and Rotterdam Study data manager (F. van Rooij, f.vanrooij@erasmusmc.nl). Local rules and regulations apply. This process includes submitting a research proposal to the RS management team and upon approval analysis needs to be performed on a local server with protected access, complying with GDPR regulations.
