# Supplementary material for: A comparison of genotyping arrays
Source: Eur J Hum Genet. 2021 Jun 18;29(11):1611–24. doi: 10.1038/s41431-021-00917-7 (PMC8560858; doi:10.1038/s41431-021-00917-7)
Supplement: Supplementary file 1 — Supplementary figure legends [file 41431_2021_917_MOESM1_ESM.docx]

# Supplementary Figure legends

Supplementary Figure 1: Examples of high and low quality clusters. Low quality clusters are generally excluded.

Supplementary Figure 2: Estimated genome-wide coverage of each genotyping array in A) EUR, B) ASN and C) AFR populations. The arrays are ordered based on number of variants they contain. Arrays with fewer variants at the bottom of the graph. Arrays in blue are from Illumina, while arrays in red are from Affymetrix.

Supplementary Figure 3: Imputation quality (1KGPp3v5) of HapMap European Ancestry samples for 6 different arrays.

Supplementary Figure 4: Imputation quality (1KGPp3v5) of HapMap Asian Ancestry samples for 6 different arrays.

Supplementary Figure 5: Imputation quality (1KGPp3v5) of HapMap African Ancestry samples for 6 different arrays.

Supplementary Figure 6: Concordance between imputed genotypes (HRC1.1) and exome sequencing genotypes for RS1. The Box plots represent the median and interquartile range

Supplementary Figure 7: Imputation quality (HRC1.1) of RS1 for 4 different arrays.

Supplementary Figure 8: Percentage of variants from the GWAS catalog covered by the arrays either directly genotyped or after imputations with good imputation quality (R2>0.8). Arrays are ordered from small to large, with the smallest array at the bottom of the graph. The imputed datasets are plotted at the top of the graph

Supplementary Figure 9: counts of number of variants in A) mtDNA, B) mtDNA divided by features, C) Actionable genes and D) pharmacogenetics genes. Arrays are ordered by number of variants from small at the bottom to large at the top. Arrays in blue are from Illumina, while arrays in red are from Affymetrix. The Box plots (B-D) represent the median and interquartile range

Supplementary Figure 10: Percentage covered of CYP450 *-alleles. Arrays are ordered on size with the smallest array shown on the bottom. Imputed datasets are placed on the top. Arrays in blue are from Illumina, while arrays in red are from Affymetrix.

Supplementary Figure 11: Percentage covered of HLA *-alleles. Percentage covered of each of the class I (figure A-C) and class II (figure D-F) genes presented. Percentages were calculated separately for EUR, ASN and AFR ancestry samples due to different tagging SNPs. Arrays are ordered on size with the smallest array shown on the left. Imputed datasets are placed on the right.

Supplementary Figure 12: Distribution of distance between SNPs within the coding regions of each gene, averaged per gene. Arrays are ordered from smallest to largest from bottom to top. Arrays in blue are from Illumina, while arrays in red are from Affymetrix. The Box plots represent the median and interquartile range
