# Supplementary figures and images for: A comparison of genotyping arrays
Source: Eur J Hum Genet. 2021 Jun 18;29(11):1611–24. doi: 10.1038/s41431-021-00917-7 (PMC8560858; doi:10.1038/s41431-021-00917-7)

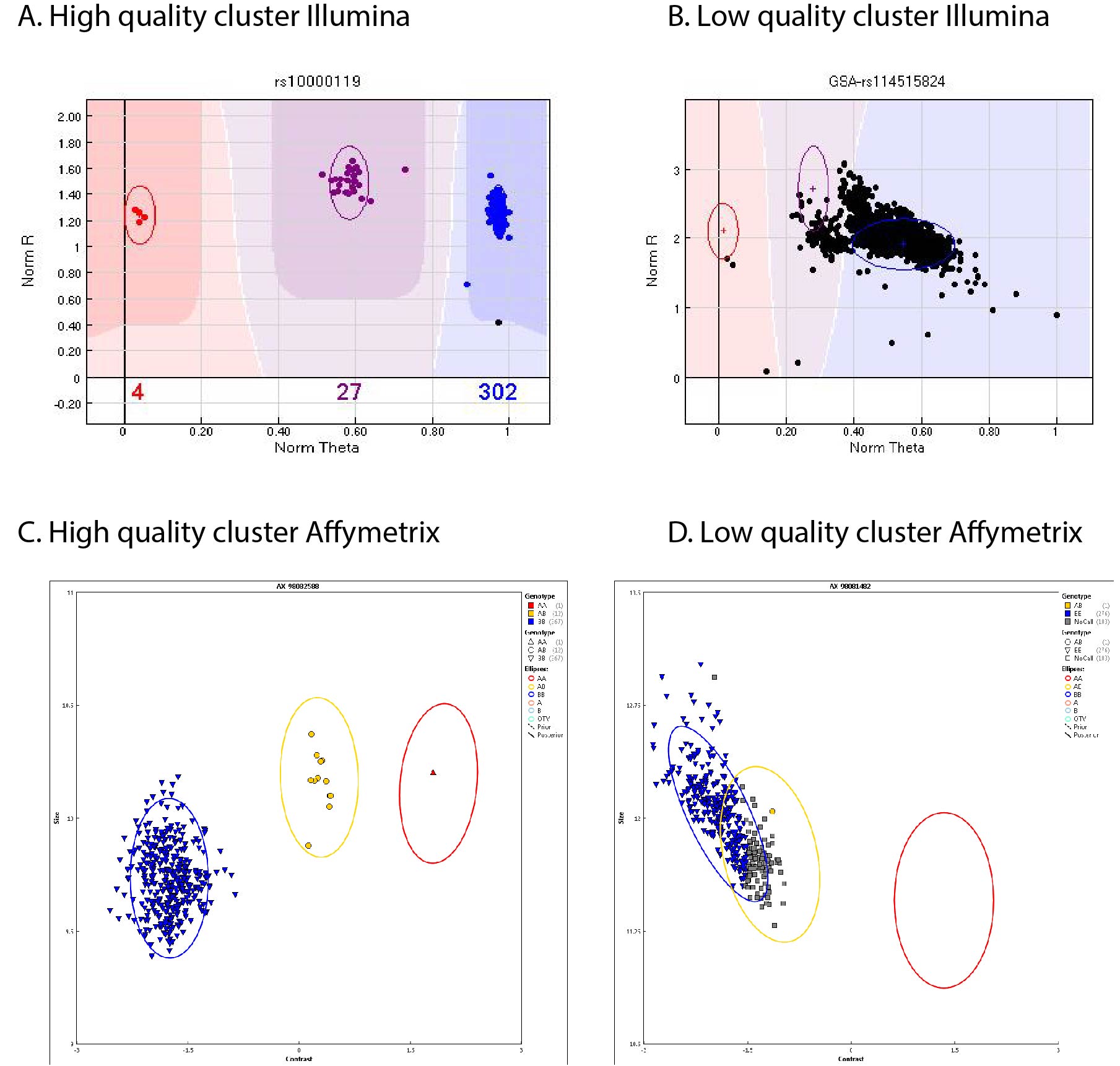

Supplement: Supplementary file 2 — Supplementary Figure 1 [file 41431_2021_917_MOESM2_ESM.jpg]

# Genome-wide coverage

## A. EUR

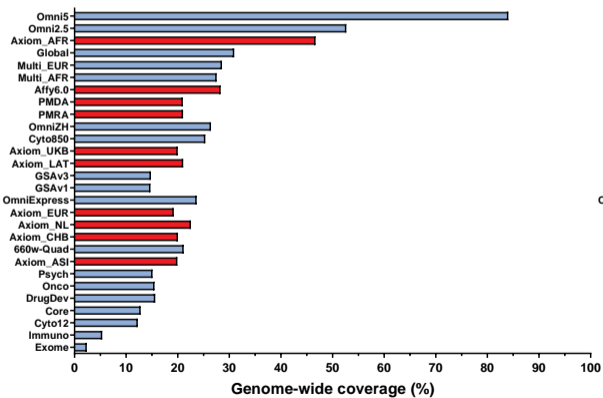

## B. ASN

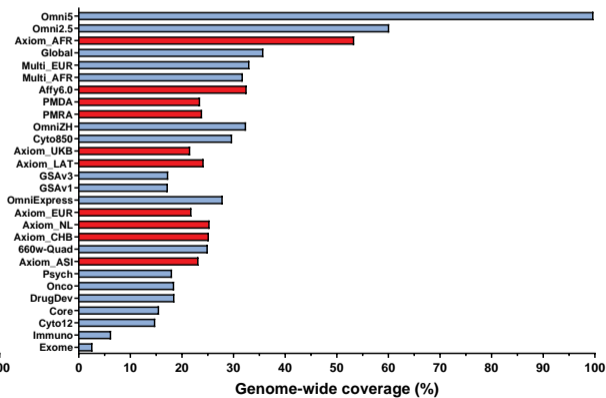

## C. AFR

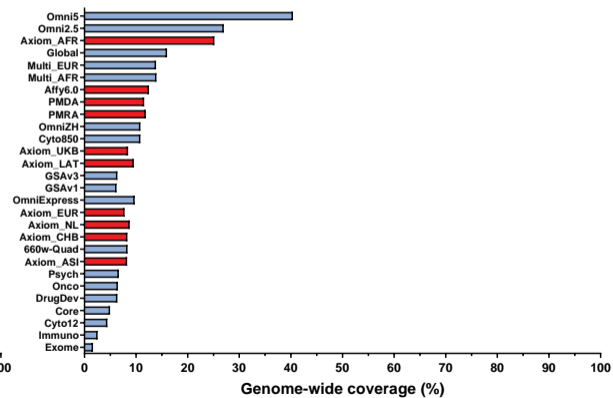

Supplement: Supplementary file 3 — Supplementary Figure 2 [file 41431_2021_917_MOESM3_ESM.pdf]

# HapMap EUR samples (N=90)

## A. Ultra-rare (MAF<0.5%)

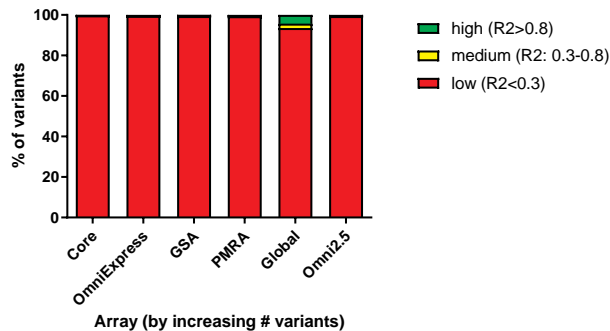

## B. Rare (MAF 0.5-1%)

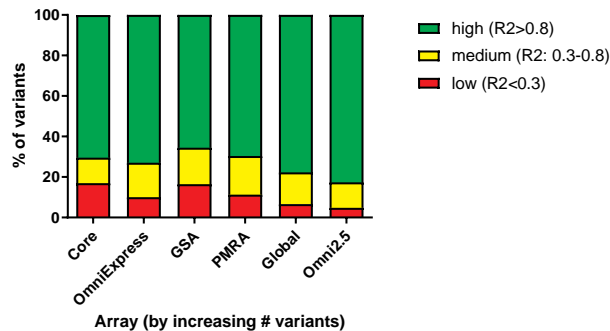

## C. Low-frequency (MAF 1-5%)

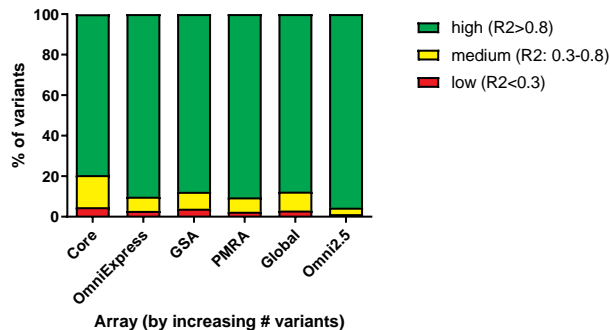

## D. Common (MAF > 5%)

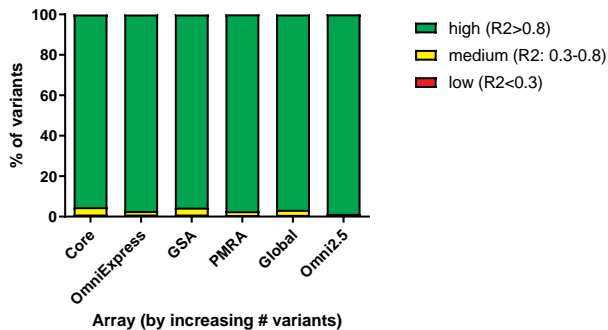

Supplement: Supplementary file 4 — Supplementary Figure 3 [file 41431_2021_917_MOESM4_ESM.pdf]

# HapMap ASN samples (N=90)

## A. Ultra-rare (MAF<0.5%)

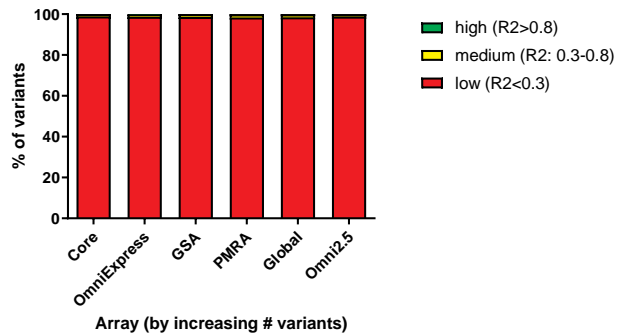

## B. Rare (MAF0.5-1%)

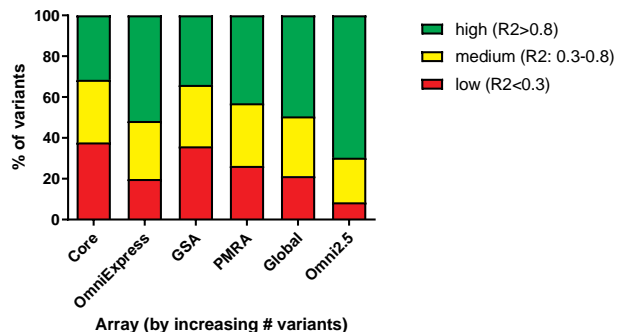

## C. Low-frequency (MAF1-5%)

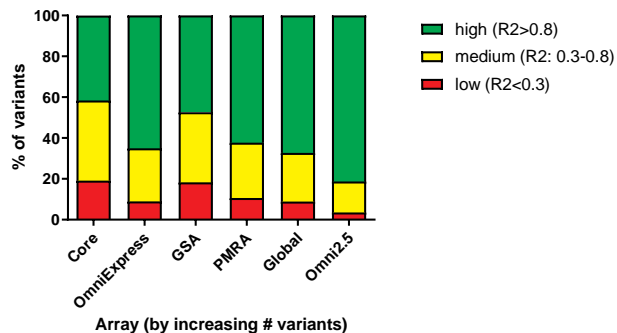

## D. Common (MAF>5%)

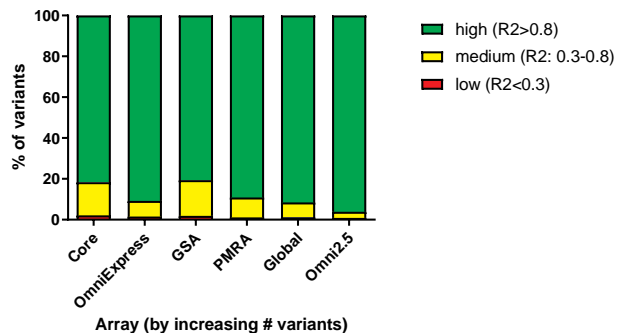

Supplement: Supplementary file 5 — Supplementary Figure 4 [file 41431_2021_917_MOESM5_ESM.pdf]

# HapMap AFR samples (N=90)

## A. Ultra-rare (MAF<0.5%)

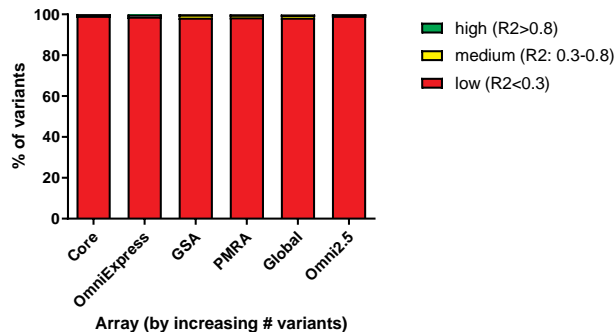

## B. Rare (MAF 0.5-1%)

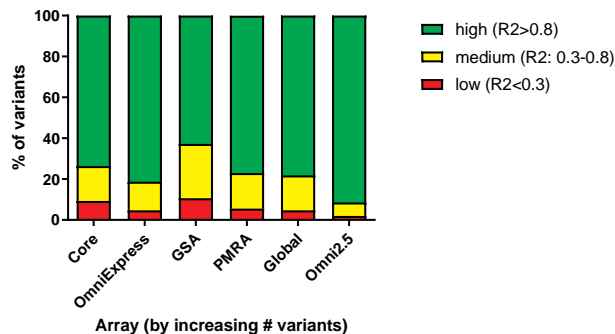

## C. Low-frequency (MAF 1-5%)

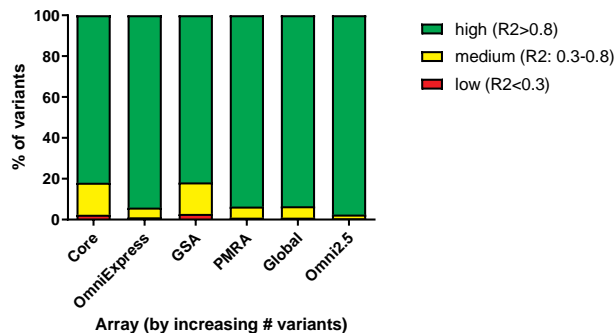

## D. Common (MAF > 5%)

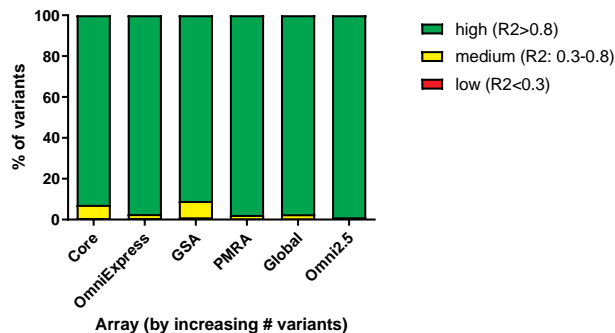

Supplement: Supplementary file 6 — Supplementary Figure 5 [file 41431_2021_917_MOESM6_ESM.pdf]

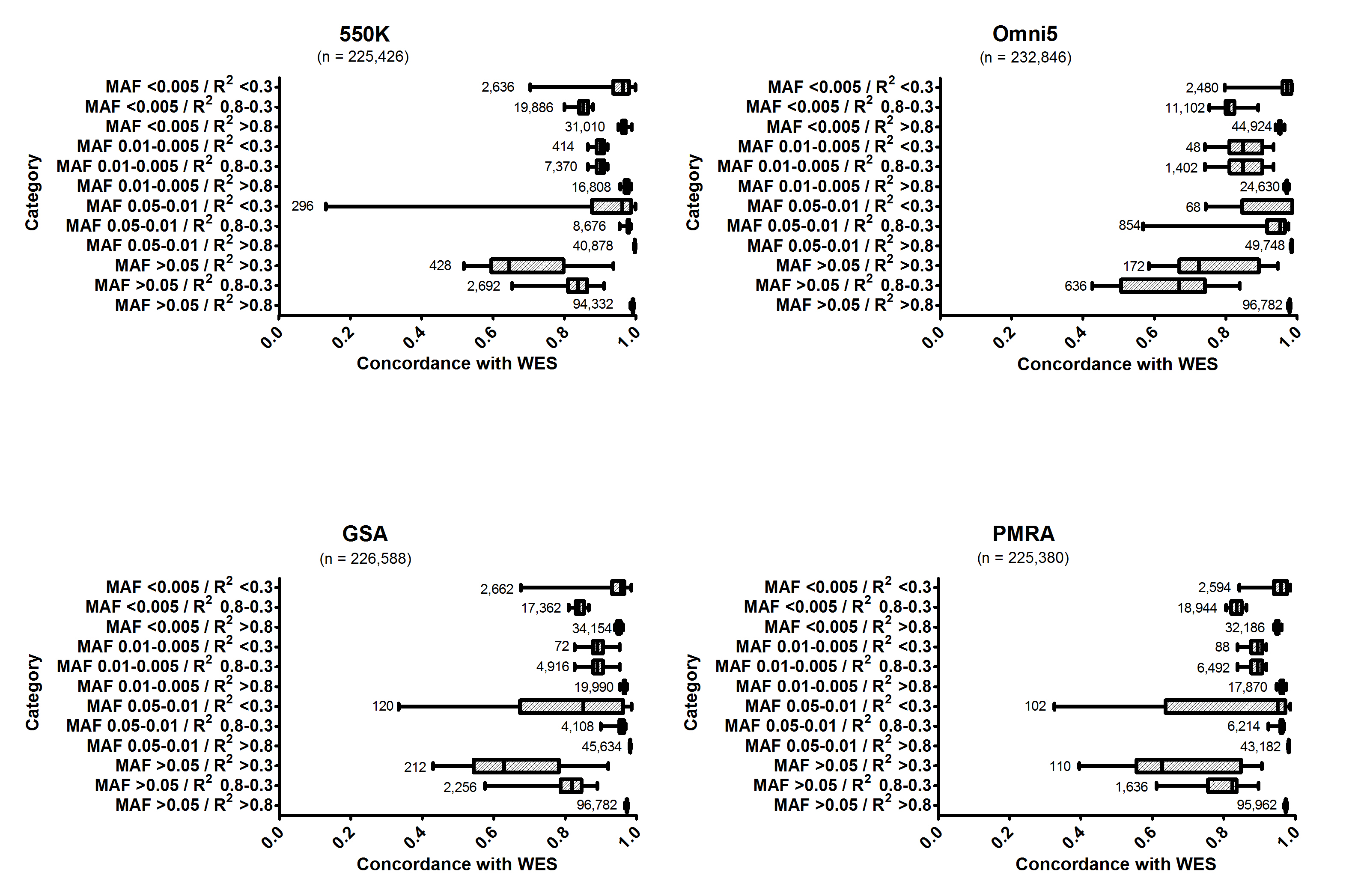

Supplement: Supplementary file 7 — Supplementary Figure 6 [file 41431_2021_917_MOESM7_ESM.jpg]

# RS-I (N=374)

## A. Ultra-rare (MAF<0.5%)

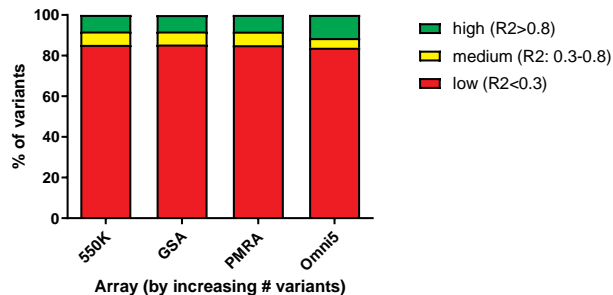

## B. Rare (MAF0.5-1%)

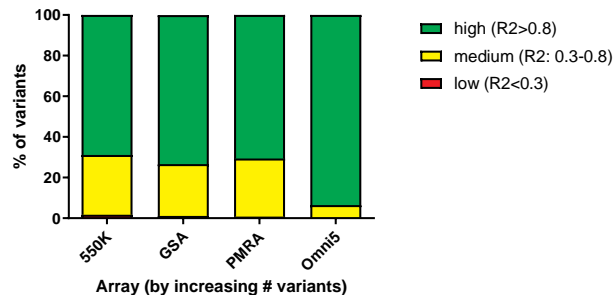

## C. Low-frequency (MAF1-5%)

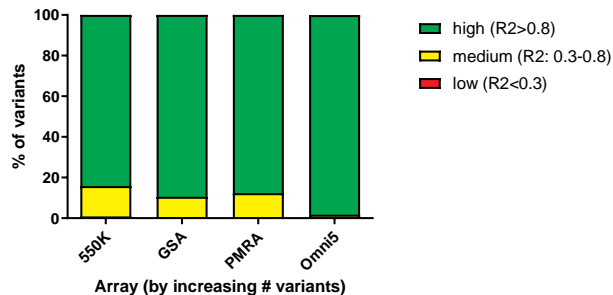

## D. Common (MAF>5%)

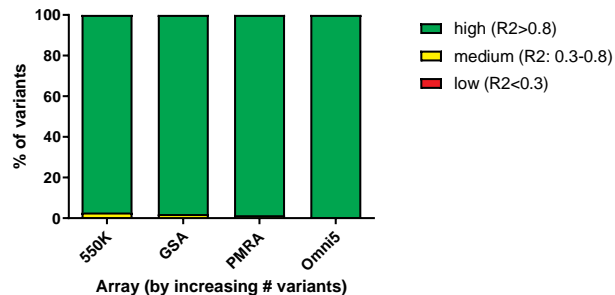

Supplement: Supplementary file 8 — Supplementary Figure 7 [file 41431_2021_917_MOESM8_ESM.pdf]

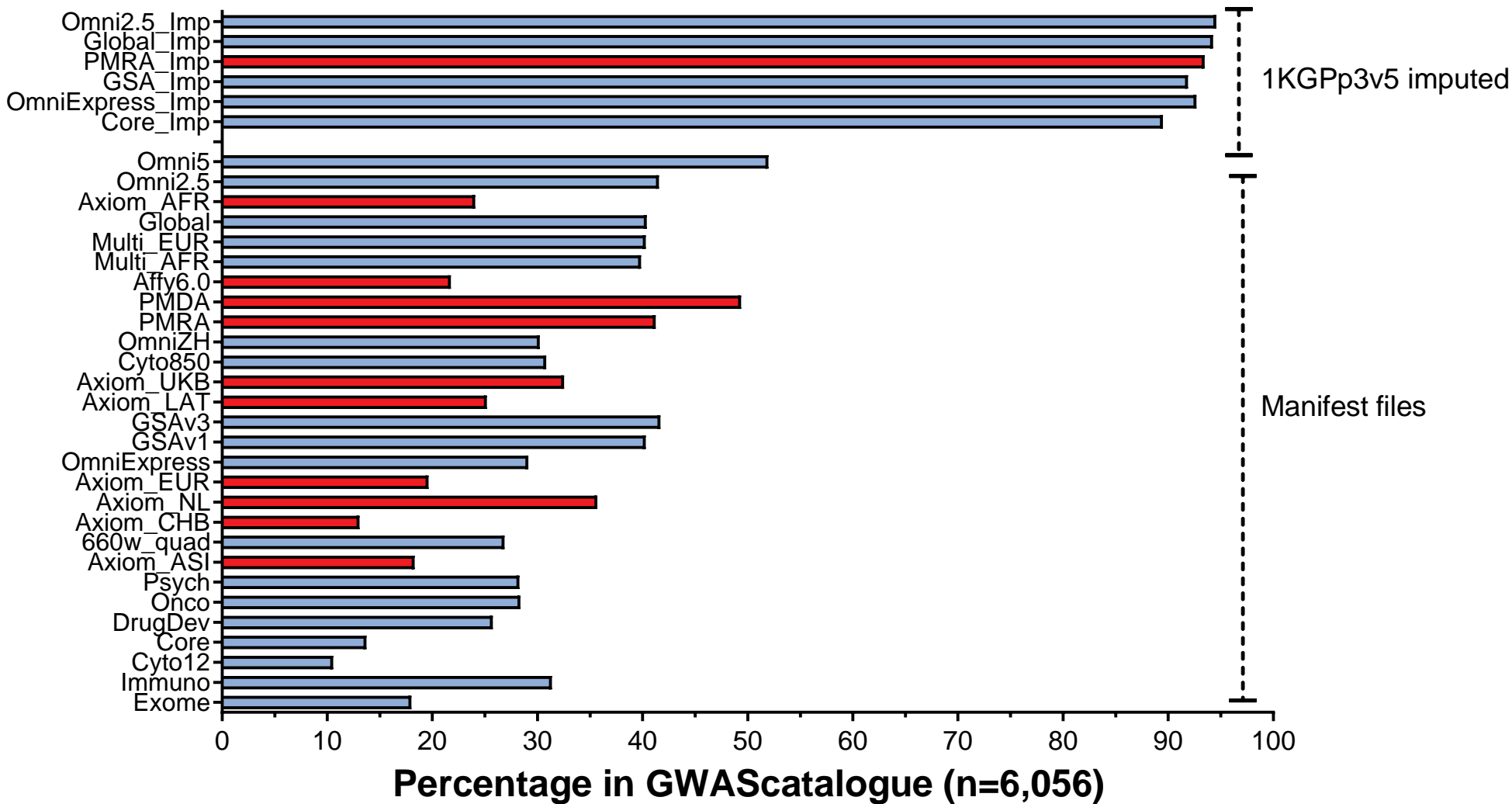

Supplement: Supplementary file 9 — Supplementary Figure 8 [file 41431_2021_917_MOESM9_ESM.pdf]

## A. mtDNA total

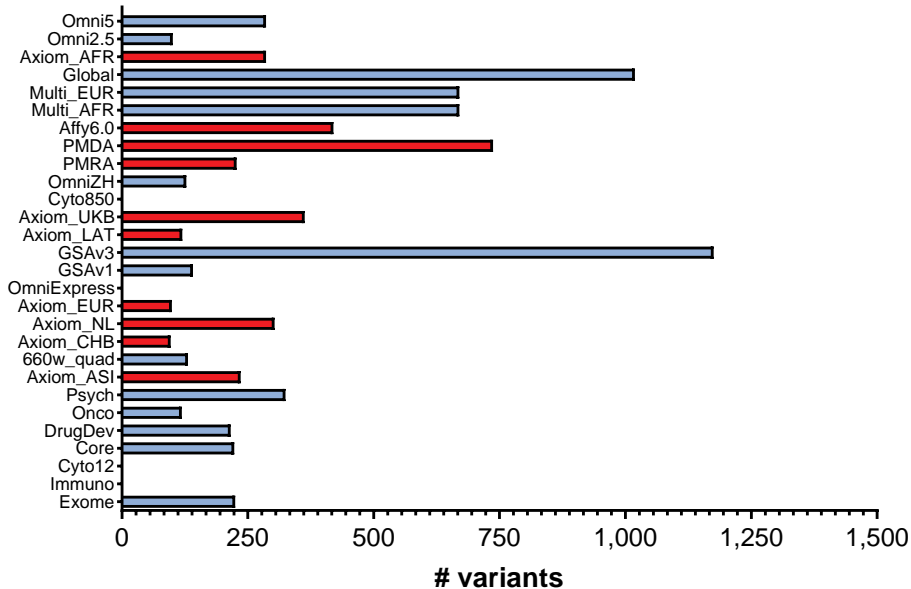

## B. mtDNA per feature

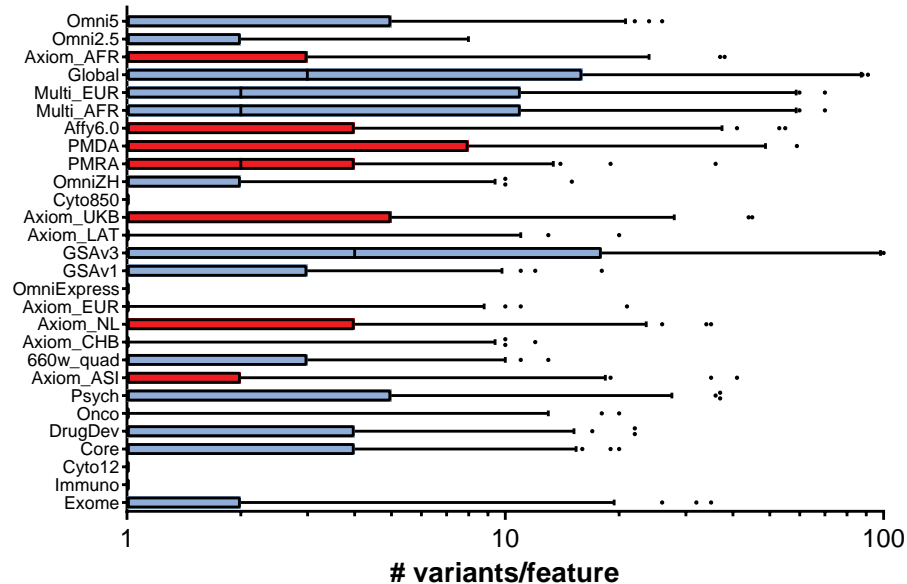

## C. Actionable Genes

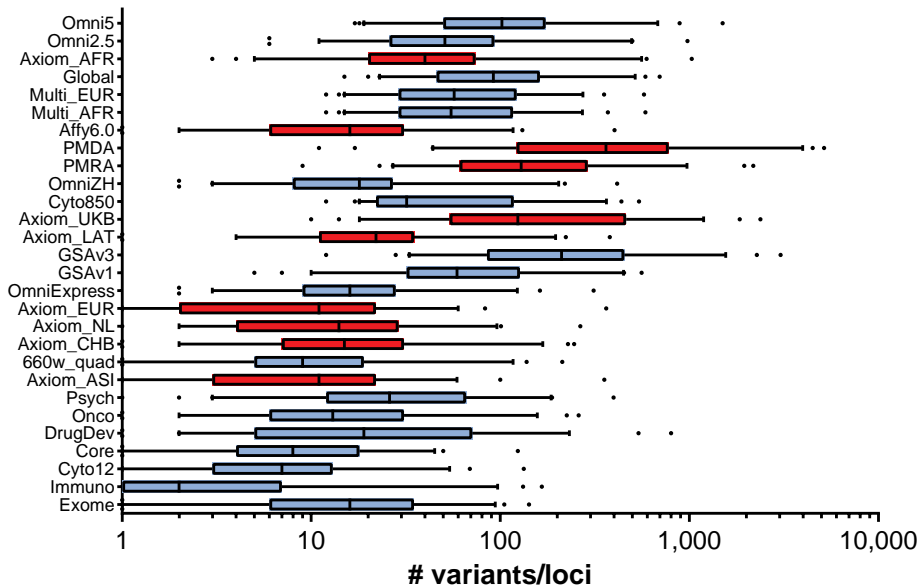

## D. Pharmacogenetics

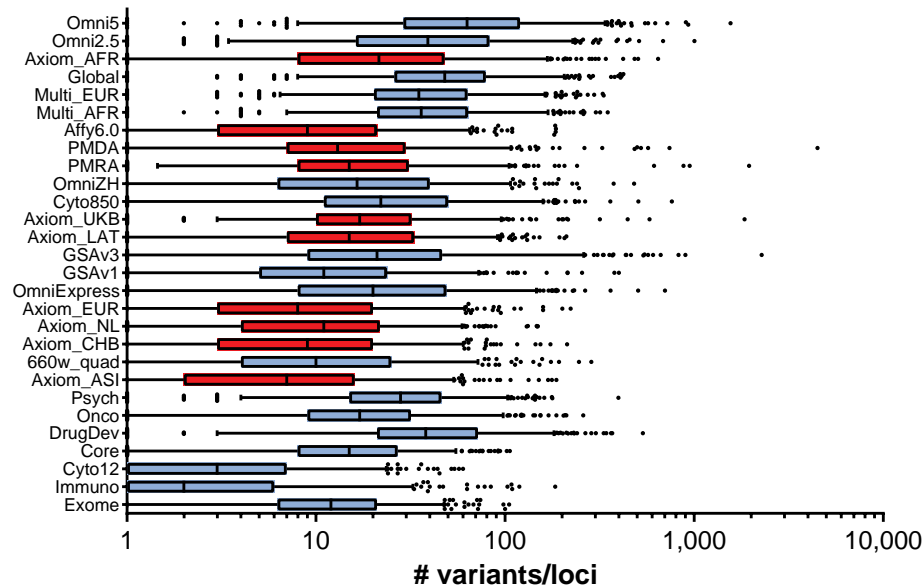

Supplement: Supplementary file 10 — Supplementary Figure 9 [file 41431_2021_917_MOESM10_ESM.pdf]

## A. CYP1A1

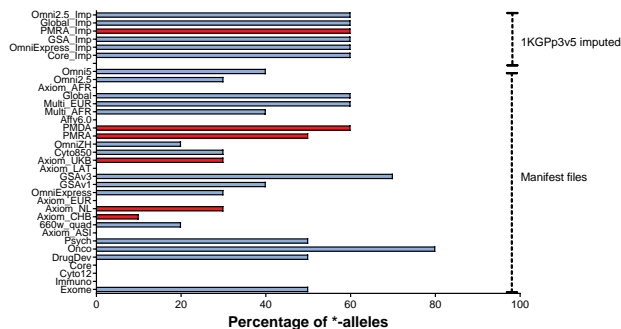

## B. CYP1A2

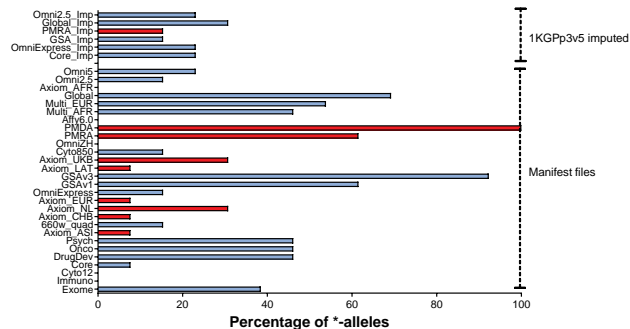

## C. CYP2A6

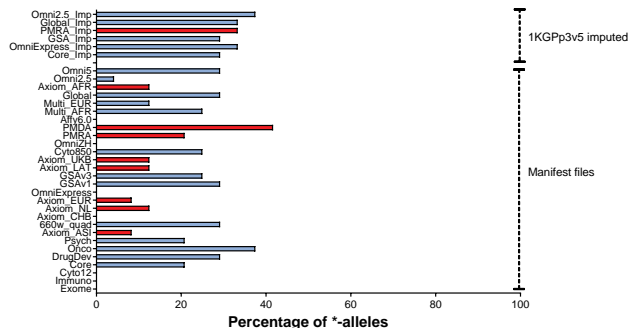

## D. CYP2B6

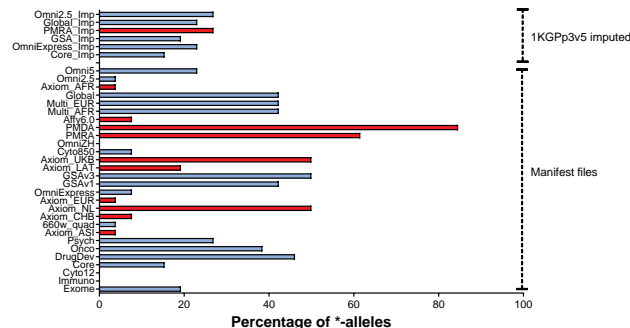

## E. CYP2C19

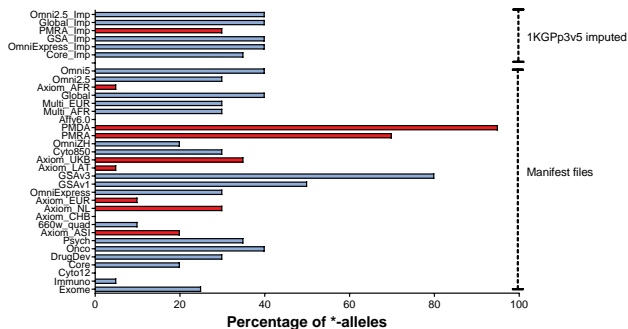

## F. CYP2C8

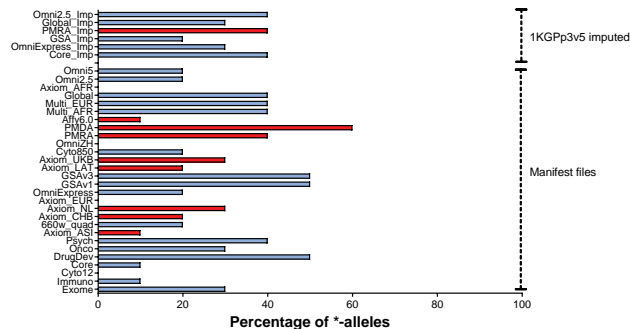

Supplement: Supplementary file 11 — Supplementary Figure 10a [file 41431_2021_917_MOESM11_ESM.pdf]

## G. CYP2C9

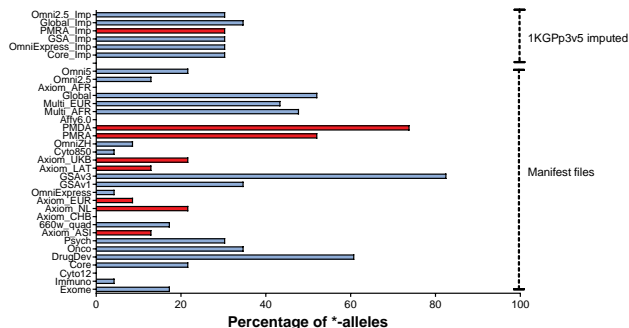

## H. CYP2D6

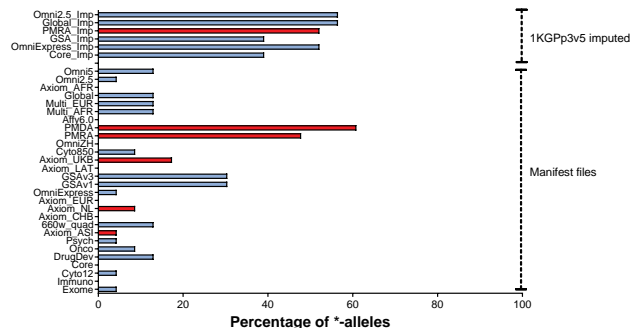

## I. CYP2E1

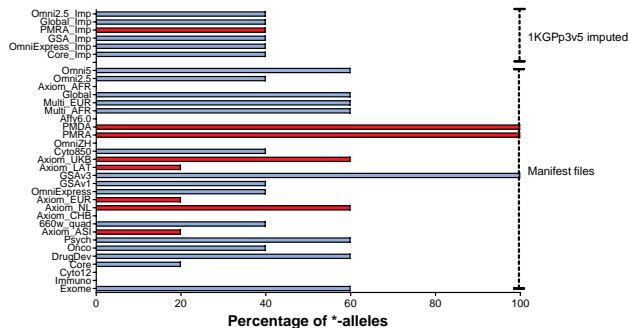

## J. CYP3A4

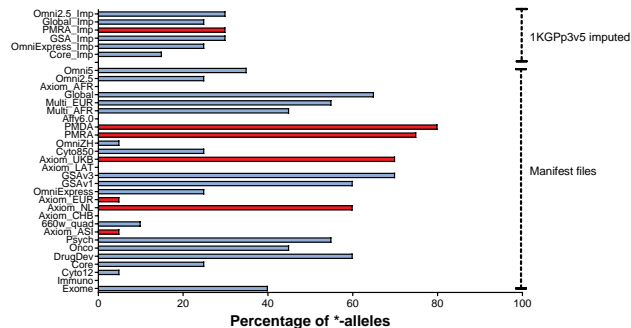

## K. CYP3A5

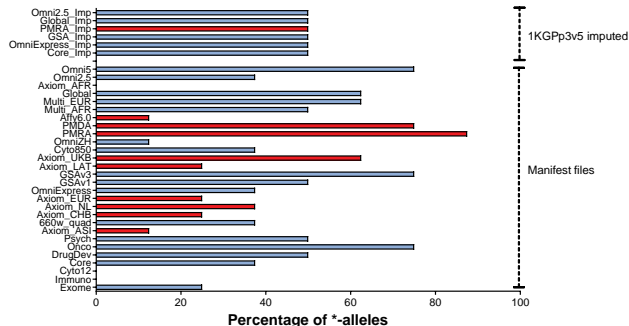

## L. CYP4F2

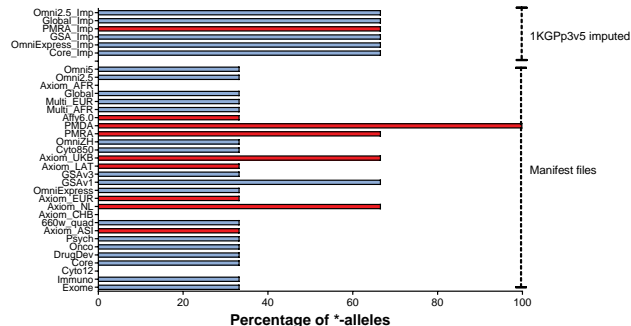

Supplement: Supplementary file 12 — Supplementary Figure 10b [file 41431_2021_917_MOESM12_ESM.pdf]

A. HLA-A

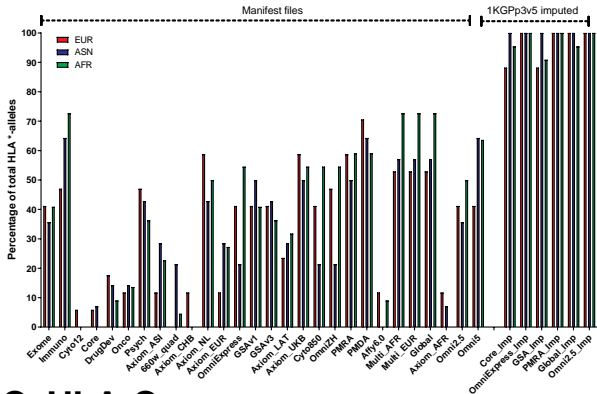

B. HLA-B

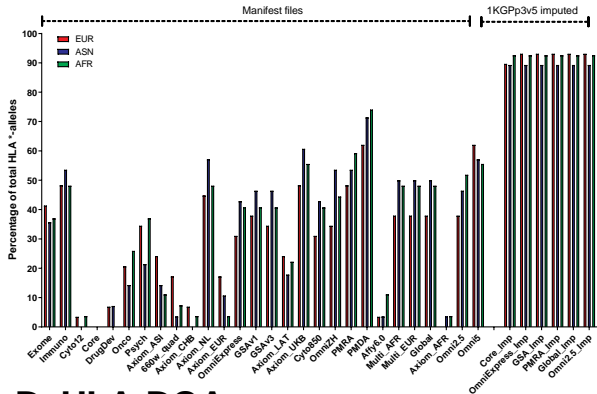

C. HLA-C

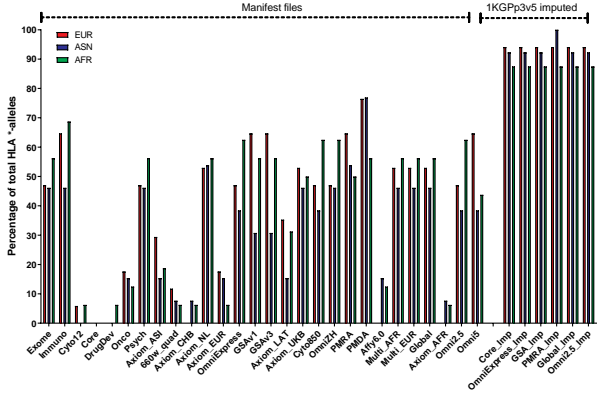

D. HLA-DQA

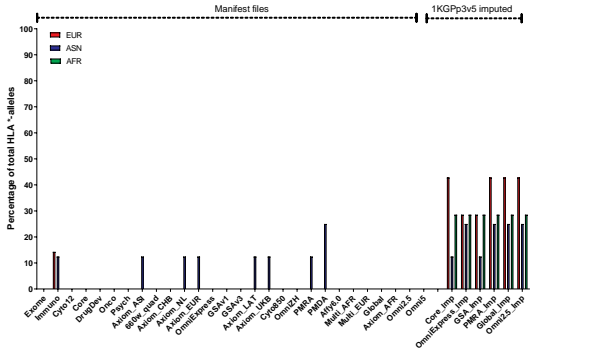

E. HLA-DQB

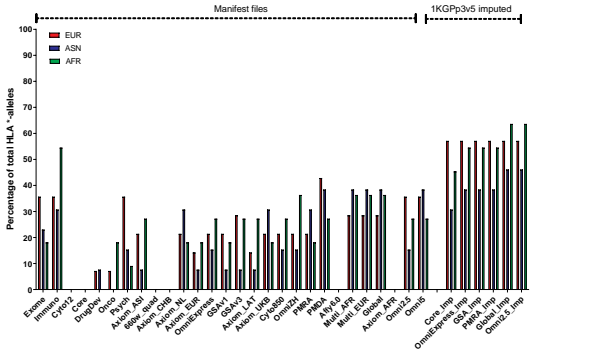

F. HLA-DRB

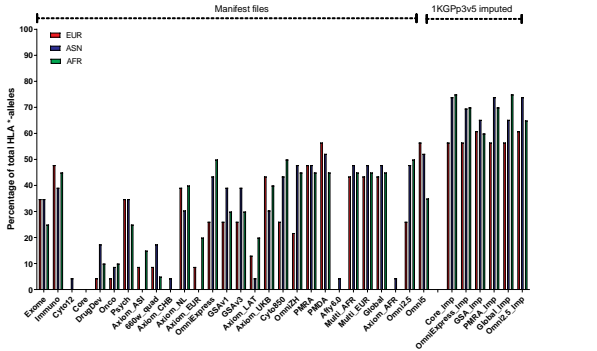

Supplement: Supplementary file 13 — Supplementary Figure 11 [file 41431_2021_917_MOESM13_ESM.pdf]

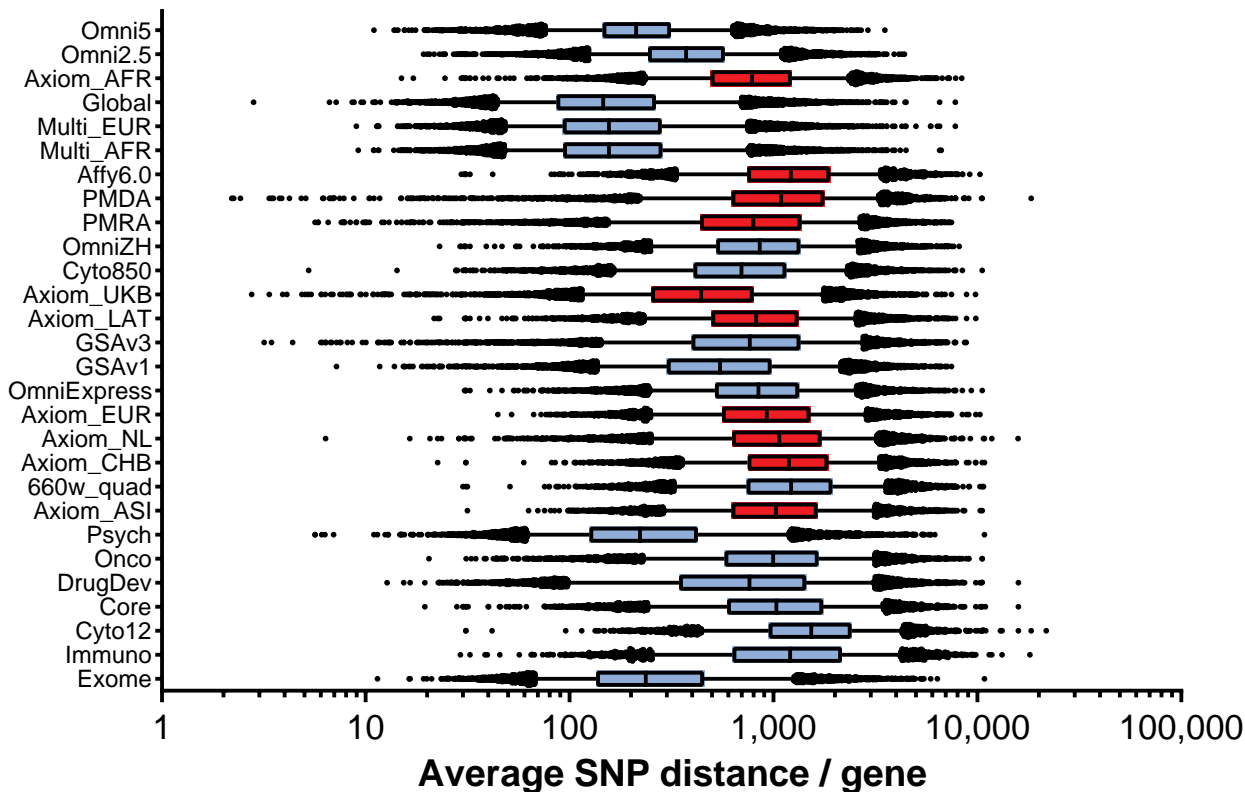

Supplement: Supplementary file 14 — Supplementary Figure 12 [file 41431_2021_917_MOESM14_ESM.pdf]
